# Knowledge, Attitude, and Practice towards Hepatitis B Virus among Pregnant Women Attending Antenatal Care at the University of Gondar Comprehensive Specialized Hospital, Northwest Ethiopia

**DOI:** 10.1155/2020/5617603

**Published:** 2020-01-15

**Authors:** Teklay Gebrecherkos, Getu Girmay, Mulualem Lemma, Markos Negash

**Affiliations:** ^1^Department of Medical Microbiology, School of Biomedical and Laboratory Sciences, College of Medicine and Health Sciences, University of Gondar, Ethiopia; ^2^Department of Medical Microbiology and Immunology, School of Medicine, College of Health Sciences, Mekelle University, Ethiopia; ^3^Department of Immunology and Molecular Biology, School of Biomedical and Laboratory Sciences, University of Gondar, Ethiopia

## Abstract

**Background:**

Hepatitis B virus (HBV) infection remains a serious public health concern worldwide. Mother-to-child transmission (MTCT) is the major mode in endemic areas, including Ethiopia, where little is known about pregnant women's knowledge, attitudes, and practice towards HBV infection and MTCT. Therefore, the study is aimed at determining the knowledge, attitude, and practice towards HBV among pregnant women attending antenatal care.

**Method:**

A cross-sectional study was conducted from February to April 2018, at the University of Gondar Comprehensive Specialized Hospital. A total of 354 pregnant women were selected by systematic random sampling and included in this study. KAP of participants on HBV MTCT was assessed using a structured questionnaire. Data was analyzed using SPSS version 22 software.

**Result:**

The total response rate was 100% (354/354). Out of the 354 participants, 73.4% were within the poor knowledge. Only 18.9% of the respondents know HBV can be transmitted from mother to child during pregnancy. Less than half (43.8) of the participants think that they will never be infected with HBV, and 47.7% of them go to traditional healers when they have symptoms of HBV. Majority of the respondents (85.87%) had never screened for HBV, and only 28.5% of the participants believed that hepatitis B can cause liver cancer. In multivariable analysis, residence, income, and educational level were associated with mean score knowledge and attitude.

**Conclusions:**

Knowledge about HBV among pregnant women was found to be poor, and their attitude and practice were also limited. Therefore, extensive health education program should be given to the pregnant women to increase their awareness towards HBV infection. All pregnant women should be screened for HBV as part of ANC follow-up.

## 1. Introduction

Hepatitis B virus (HBV) is a deoxyribonucleic acid (DNA) virus belonging to a family Hepadnaviridae that causes acute or chronic infection [1]. HBV infection is the 10^th^ leading cause of death resulting 500,000 to 1.2 million deaths per year, with 2 billion people infected worldwide and 257 million suffering from chronic HBV infection, of which 10% of these are in sub-Saharan Africa and East Asia [2, 3].

HBV affects all age groups globally including pregnant woman and the newly born infant vertically. The global prevalence of HBV among pregnant women and rate of vertical transmission greatly vary from continent to continent [4, 5].

Several ways of HBV transmission are registered including contaminated blood transfusion, unprotected sex, and streak with contaminated sharp objects [6]. Mother-to-child transmission (MTCT), by which HBV is transmitted from infected mothers to their infants, prenatal transmission (in utero), natal transmission (during delivery), or postnatal transmission (during childcare or through breast milk, is the main route of infection in infants [7, 8]. Following HBV infection, many people with HBV may not show any symptoms and the clinical manifestations vary in acute and chronic cases from nonspecific symptoms to organ failure [6, 9].

Hepatitis B virus is a life-threatening infection, and the prevalence of HBV infection varies widely, with rates ranging from 0.1–20% in different parts of the world. The prevalence of HBV in the pregnant women in the Asian region is variable with the highest rates in Taiwan (>10%) and Thailand (>8%) and the lowest in Japan (0.8%), with the majority of countries having rates less than 8% [10].

Prevalence of HBeAg positivity varies widely among HBsAg-positive pregnant women: less than 2% in Ethiopia, Ghana, and Nigeria; 3.3% in Zimbabwe; 4.6% in South Africa; 9.5% in Senegal; 16.1% in Zambia; and 24% in southern Tanzania. Compared with pregnant chronic HBsAg-infected women in the other parts of the world where HBV is hyperendemic (Southeast Asia), those in Africa have a low rate of HBeAg positivity [4].

A study conducted in eastern Ghana in 2016 showed that 59.8% pregnant women had poor knowledge, 64.7% of them had negative attitude, and 73.7% of them had poor practice towards HBV in the study. This revealed a poor level of knowledge, attitude, and practice (KAP) among an average of 66.1% pregnant women in the study [11], while a study conducted in Nigeria showed that only 75.2% antenatal care- (ANC-) attending women do not know that hepatitis is a viral infection affecting the liver [12].

On the other hand, a study conducted in Ghana showed that only 49% of respondents knew that HBV can be transmitted through blood and 42.8% of the respondents knew that unprotected sex could lead to HBV transmission [13]. One previous study conducted in Addis Ababa, Ethiopia, revealed that 60.8% pregnant women were within the poor knowledge range, 18.9% respondents said that transmission could occur from mother to child during pregnancy, and 57.3% of pregnant women showed negative practice [14].

In the presence of high magnitude, rapid rate of transmission, and severe complications including death in pregnant women and infants, the infection is still prevalent [13]. Among pregnant women, these illnesses can lead to coagulation defects, postpartum hemorrhage, organ failure, and high maternal death and poor outcomes of their newborns such as stillbirths, neonatal deaths, acute and chronic liver disease, and hepatocellular carcinoma [14]. However, studies conducted to assess the KAP towards HBV among pregnant women in Ethiopia are limited and such studies have never been conducted in our study area. Assessing the KAP among pregnant women is the best way of HBV infection prevention, which ultimately will reduce vertical transmission by giving health education. In addition, results obtained from this study are important to program managers and health planners, to plan vaccination and other preventive strategies. Thus, this study is aimed at assessing the knowledge, attitude, and practice towards HBV among pregnant women attending ANC at the University of Gondar Comprehensive Specialized Hospital, Northwest Ethiopia.

## 2. Methods and Materials

### 2.1. Study Design, Area, and Period

An institution-based cross-sectional study was conducted from February to April 2018 at the Gondar University Hospital (GUH), Gondar Town, Northwest Ethiopia. It is a comprehensive specialized and teaching hospital, which is located in Gondar Town, Amhara National Regional State, and 750 km far from Addis Ababa (capital city of Ethiopia) in Northwest Ethiopia. The hospital has served more than 5 million people who live in North Gondar Zone and people of the neighboring zone. The hospital consists of an operating room, antenatal care, intensive care unit (ICU), fistula center, 13 different wards, and outpatient departments.

### 2.2. Study Population, Sample Size, and Sampling Technique

All pregnant women attending the ANC clinic in the University of Gondar Comprehensive Specialized Hospital during the study period were included. The sample size was determined using the following single-population proportion formula: *N* = *z*^2^ *p* (1 − *p*)/*w*^2^, where *N* is the number of pregnant women attending the ANC clinic, *z* is the standard normal distribution value at 95% CI which is 1.96, *p* is the prevalence of having good practice among pregnant women (42.7%, previous prevalence report from Addis Ababa) [14], and *w* is the margin of error, taken as 5%. Accordingly, the sample size calculated was 354.

A systematic random sampling technique was used by considering that the ANC clinic in the University of Gondar Comprehensive Specialized Hospital on average gave 25 pregnant women per day so that data collection was planned for 3 months: *k* = *N*/*n* = 2250/354 = 7. Then, every 7 pregnant women that come to the ANC clinic from February to April was included in the sample until the required sample was achieved.

### 2.3. Operational Definition

*Knowledge*: information stored in memory assessed in terms of what the participants know about HBV.

*Knowledgeable*: study participants who correctly answered greater than or equal to 70% of knowledge-related questions.

*Not knowledgeable*: study participants who answered less than 70% of knowledge-related questions.

*Attitude*: complex interaction of beliefs, feelings, and values to respond in a manner towards HBV.

*Positive attitude*: study participants who answered correctly greater than or equal to 70% of the attitude-related questions about HBV.

*Negative attitude*: study participant who answered less than 70% of the attitude-related questions about HBV.

*Practice*: what the respondents actually practice for prevention and control of HBV.

### 2.4. Data Collection Tool and Technique

Sociodemographic characteristics (age, sex, educational background, occupation, residence, and income) and variables related to KAP like source of information, screening for HBV, accessibility of health service, availability of vaccine, and sharing of needles and blades were gathered using a pretested structured questionnaire.

### 2.5. Quality Control

The questionnaire was originally prepared in English and translated into Amharic and back to English to keep the consistency of the questions by independent individuals. Training of the data collection team was made to insure the possible quality data. All authors checked and reviewed the filled questionnaires to ensure completeness and consistency of the information collected.

### 2.6. Data Analysis

Data were double entered and analyzed by using SPSS-20 database software program. Categorical variables were measured as percentages while continuous variables were expressed as mean ± standard deviation. The Kolmogorov-Smirnov test was applied to declare the nature of data distribution. Frequencies were used to summarize descriptive statistics; a crosstabulation was used to relate between dependent and independent variables.

Knowledge was assessed by questions focusing on HBV etiology, sign and symptoms, transmission, and treatment. Each response was scored as “yes” or “no.” The scoring range of the questionnaire was 1 to 11. A cutoff level ≤ 5 was considered poor whereas >5 was considered adequate knowledge about HBV. Knowledge scores for individuals were calculated and summed up to give the total knowledge score.

Practices towards HBV were assessed by asking five questions listed in Table 1. Each question was labeled with good or poor practice. A score of 1 was given to good while 0 was given to bad practice with a score range of 0 to 5. The scale classified practice as positive with score > 3 and negative ≤3.

### 2.7. Ethical Consideration

Ethical approval was obtained from the School of Biomedical and Laboratory Sciences Ethical Review Committee. Permission was obtained from the University of Gondar Comprehensive Specialized Hospital. The purpose and importance of the study was explained to each study participant. To ensure the confidentiality of participant's information, anonymous typing was used whereby names of the participants and any other identifiers were excluded from the questionnaire. For further purposes of privacy, participants were interviewed separately. Above all, data was collected after a full verbal consent was obtained from each participant.

## 3. Results

### 3.1. Sociodemographic Characteristics

A total of 354 pregnant women with 100% response rate from the ANC clinic were enrolled during the study. The mean age was 25 years (SD ± 10.4), and the median age was 30 (IQR: 15 to 49) years. The educational status tally showed that 94 (26.6%) were unable to read and write, while 22% attended high school and above. 251 (71%) of the participants had a monthly income of less than five hundred birr. In addition, sixty-five (54.5%) of them were found to be housewives (Table 2).

### 3.2. Assessment of Knowledge towards HBV

Knowledge scores for individuals were summed to divide the participants into having adequate knowledge (answered >5 knowledge-related questions correctly) and having poor knowledge (who answered ≤5 of knowledge-related questions).

Out of the 354 participants, 260 (73.4%) were within the poor knowledge range whereas 94 (26.6%) showed good knowledge.

According to the present findings, 244 (68.9%) of the participants did not know the availability of vaccine against HBV, while 293 (82.8%), 289 (81.6), and 304 (85.8%) of them did not know the transmission way of HBV from mother to child, through contaminated blood, and through unsafe sex, respectively. Moreover, 243 (68.7%) of the participants responds HBV affects liver (Table 3).

### 3.3. Assessment of Attitude towards HBV

Each question was labeled with positive or negative attitude. A score of 1 was given to positive while 0 was given to negative attitudes with a score range of maximum of 8 to a minimum of 0. Of the total participants, 191 (54%) were having positive attitude (who answered ≥5 related questions correctly) and 163 (46%) were having negative attitude (who were giving answer of <5 for attitude-related questions). Most of the participants (155, 43.8%) think that they will never be infected with HBV, and 169 (47.7%) of them go to traditional healers when they have symptoms of HBV (Table 4).

### 3.4. Assessment of Practice towards HBV

Practices towards HBV were assessed by asking five questions as shown in Table 1. Each question was labeled with good or poor practice. A score of 1 was given to good while 0 was given to poor practice with a score range of maximum of 5 to a minimum of 0. The scale classified practice as good with score > 3 and poor ≤3. Out of the 354 participants, 282 (79.7%) were within the poor practice range while 72 (20.3%) showed good practice. Majority of the respondents (304, 85.87%) had never screened for HBV, and 346 (97.7%) are not immunized against HBV, while 182 (51.4%) of them did not ask their barber to change the blade for safe equipment for ear and nose piercing.

### 3.5. Association of Demographic Characteristics and Mean KAP Scores

The association of demographic characteristics and mean KAP scores is presented in Table 5. Among the demographic variables, area of residence, educational status, and income were significantly associated with mean KAP scores (*P* < 0.001). On the other hand, religion was significantly associated with attitude and practice (*P* < 0.001), while occupation was only associated with practice (*P* < 0.001).

### 3.6. The Relation between Knowledge and Practice Responses with Sociodemographic Characteristics

As shown in Table 6, the majority especially who had poor knowledge and practice responded negatively. More than half of the women (190, 53.7%) and 204 (57.6%)) with poor knowledge and practice were obtained among urban residents, respectively, please delete this?. Moreover, 196 (54.8%) and 216 (61%) that responded poor knowledge and practice had less than 500 ETB monthly income.

Those women with poor knowledge for HBV were 2.3 times more likely to live in urban areas than the corresponding rural dwellers (AOR = 2.3; 95% CI, 1.2-4.4; *P* = 0.012).

Those respondents with monthly income of less than 500 ETB were 1.7 times more likely to be higher among women with poor knowledge compared with good knowledge (AOR = 1.7, 95%CI = 1.4, 3.0; *P* = 0.05), while women with monthly income of less than 500 ETB were 2.5 times more likely to respond poor practice (AOR = 11.28, 95%CI = 2.25, 56.7; *P* = 0.03) than the corresponding good practice.

## 4. Discussion

The current study sought to evaluate knowledge, attitude, and practice towards HBV among pregnant women who started clinical attachment. Results of the study revealed poor knowledge and practice towards HBV. The mean knowledge score was 4.1 ± 2.6 indicating low level of knowledge towards HBV. This lack of knowledge may influence the attitudes of the mother towards interventions that could reduce the risk of transmission to their infants.

The current finding showed that 73.4% of the participants had poor knowledge, whereas another study conducted in eastern Ghana in 2016 showed that 40.2% of pregnant women had good knowledge [11]. In addition, in a cross-sectional study conducted in China in 2017, only 21% of the participants were able to answer all the general knowledge-related questions correctly [7]. Similarly, a study conducted in Ghana in 2014 revealed that less than half of the participants (46.2%) knew about hepatitis B infection and its disease [13]. Moreover, a study conducted in the Buea Health District, Cameroon, in 2012 showed that <20% of the participants had the correct knowledge [15]. On the other hand, in a study conducted in Addis Ababa, Ethiopia, in 2014, 39.2% of them had adequate knowledge about HBV [14].

According to our result where measures against HBV depend on whether people know that hepatitis is a transmissible disease or not, only 18.4% of the respondents know HBV is transmissible through blood and blood products, 14.2%% through unsafe sex, and 17.2%% from mother to child during pregnancy. This is in line with a study reported by ul Haq et al. from Pakistan [16] and Fikremariam from Addis Ababa, Ethiopia [14]. Lack of knowledge about HBV transmission can be attributed to rise in the frequency of HBV in the community. This low level of knowledge of ways of HBV transmission calls for targeted health education in order to prevent and control the spread of the virus.

On the contrary, a study conducted in Nigeria in 2015 showed that pregnant women who demonstrated good knowledge regarding the transmission of HBV from mother to child were recognized by 72.9% of respondents [12], and a study reported by Pham et al. in Vietnam in 2019 showed that 75.3% of the participants were aware that HBV is transmitted through unprotected sex [17]. Good knowledge of pregnant women in different countries regarding the different modes of transmission of hepatitis B virus infection can be explained by the fact that these women have been receiving regular antenatal care education on the subject of hepatitis B infection.

Only 28.5% of our study participants believed that hepatitis B can cause liver cancer; similarly, low level of knowledge was reported from Japan in 2013, which is 18.5% [18], from Pakistan in 2012, which is 28.2% [19], and from Adds Ababa, Ethiopia, in 2014, which is 30.5% [14]. However, it contradicts findings of Wah et al. from China who found that 87% of the study participants believed that HBV can cause liver cancer [20].

In our study, 54% of the respondents had positive attitude towards HBV. This was slightly higher than a study conducted in Honiara, Solomon Islands (35.3%) [21].

In addition, a study conducted in Bangladesh in 2012 showed that 50% of study participants had positive attitude [22]. In addition, 47.7% of our study participants had preferred traditional therapies as a treatment of choice until there is no improvement in the sign and symptoms of HBV if infected with HBV. Moreover, consulting the physicians was reported only in 22.3% of them. Would you please correct 12.2 as 11.8% and 24% as 49.2%

A study conducted in Addis Ababa, Ethiopia, in 2014 showed that 12.2 and 24% of them had gone to traditional healers as treatment of choice until there is no improvement in the sign and symptoms of HBV if infected and consulted the physicians if they are infected with HBV, respectively [14]. This difference was due to different study locations, due to low socioeconomic status, and due to being unable to communicate to physicians when infected due to limited accessibility of healthcare, availability of health facility, and low educational status of the participant.

According to this study, 20.3% of study participants had good practice. This is slightly lower than a study conducted in Honiara, Solomon Islands, in 2015 which showed that 26.3% of study participants had good practice [23]. But a high rate of good practice was reported (42.7%) from Addis Ababa, Ethiopia, in 2014 [14]. In our study, 85.9% had not screened for HBV, while a study conducted in China in 2017 showed that 68.5% of study participants had not screened [7]. This contradiction was due to limited availability of healthcare, low awareness about the availability of vaccines, not knowing the importance of screening for HBV prevention and control, and educational status of the participant.

Area of residence, unable to read and write, and income were significantly associated with mean KAP scores. Similar results were reported by ul Haq et al. from Pakistan (while income is not significantly associated with mean KAP) [16], Wu et al. in 2007 among Americans [24], and Cheung et al. in 2005 among Chinese and South Asian Canadians [25]. On the other hand, reports from Addis Ababa, Ethiopia [14], and Guangdong Province, China [7], showed that higher education level was associated with better knowledge and attitude scores. Likewise, religion was significantly associated with attitude and practice (*P* < 0.001), while occupation was only associated with practice (*P* < 0.001). However, through extensive literature review, no studies were found to report the relation of religion and occupation with mean KAP scores.

## 5. Conclusion and Recommendation

The overall knowledge of the participants was found to be poor, and their attitude and practice were also limited. In this study, most pregnant women had poor knowledge about the transmission and prevention of hepatitis B. Residence, unable to read and write, and income were significantly associated with mean KAP scores. Extensive health education campaign should be provided to the general population and especially to the residents of rural residents. Therefore, public health interventions to improve HBV antenatal screening practices are needed, particularly at primary healthcare settings, to eliminate mother-to-child transmission. Extensive health education program should be given to the pregnant women to increase their awareness towards HBV infection. All pregnant women should be screened for HBV as part of ANC follow-up.

## Figures and Tables

**Table 1:** Practice towards hepatitis B virus among pregnant women attending ANC in the University of Gondar Comprehensive Specialized Hospital, Northwest Ethiopia, in 2018.

Practice items	Response	Frequency	Percentage (%)
Have you done screening for hepatitis B?	Yes	50	14.1
	No	304	85.9
Do you ask your barber to change the blade for safe equipment for ear and nose piercing?	Yes	172	48.6
	No	182	51.4
When diagnosed with hepatitis B, would you go for further investigation and treatment?	Yes	187	52.8
	No	167	47.2
Have you got yourself vaccinated against hepatitis B?	Yes	8	2.3
	No	346	97.7
Do you avoid meeting hepatitis B patients?	Yes	29	8.2
	No	325	91.8

**Table 2:** Sociodemographic information among pregnant women attending ANC in the University of Gondar Comprehensive Specialized Hospital, Northwest Ethiopia, in 2018.

Characteristics	Response	Frequency	Percentage (%)
Age (in years)	15-26	199	56.2
	27-38	139	39.3
	39-49	16	4.5

Residence	Urban	271	76.6
	Rural	83	23.4

Marital status	Single	29	8.2
	Married	320	90.4
	Widowed	0	0
	Divorced	5	1.4

Religion	Orthodox	307	86.7
	Muslim	39	11
	Protestant	7	2
	Catholic	1	0.3

Educational status	No formal education at all	94	26.6
	Able to write and read	4	1.1
	Elementary education	99	27.96
	Secondary education	79	21.9
	College level	78	22

Occupational status	Self-employed	56	15.8
	Government employed	65	18.4
	Housewife	193	54.5
	Not employed	40	11.3

Monthly income	<500	251	71
	1000-3000	80	22.6
	3001-5000	18	5
	>5000	5	1.4

**Table 3:** Knowledge towards hepatitis B virus among pregnant women attending ANC in the University of Gondar Comprehensive Specialized Hospital, Northwest Ethiopia, in 2018.

Knowledge items	Response	Frequency	Percentage (%)
Have you heard of a disease caused by hepatitis B virus?	Yes	323	91.2
	No	31	8.8
Can hepatitis B affect liver?	Yes	243	68.7
	No	111	31.3
Can hepatitis B cause liver cancer?	Yes	101	28.5
	No	253	71.5
Are nausea, vomiting, and loss of appetite common symptoms of hepatitis B?	Yes	246	69.5
	No	108	30.5
Can hepatitis B affect all age groups?	Yes	233	65.8
	No	121	34.2
There is no symptom of hepatitis B in some patients?	Yes	75	21.8
	No	279	78.8
Can hepatitis B transmitted through contaminated blood?	Yes	65	18.4
	No	289	81.6
Can hepatitis B transmitted by blades of ear or nose pierces?	Yes	67	18.9
	No	287	81.1
Can hepatitis B transmitted by unsafe sex?	Yes	50	14.2
	No	304	85.8
Can hepatitis B transmitted by mother to child?	Yes	61	17.2
	No	293	82.8
Is hepatitis B curable/treatable?	Yes	212	59.9
	No	142	40.1
Is vaccination available for hepatitis B?	Yes	110	30.1
	No	244	68.9

**Table 4:** Attitude towards hepatitis B virus among pregnant women attending ANC in the University of Gondar Comprehensive Specialized Hospital, Northwest Ethiopia, in 2018.

Attitude items	Response	Frequency	Percentage (%)
Do you think you can get hepatitis B?	Yes	199	56.2
	No	155	43.8

What would be your reaction if you found that you have hepatitis B?	Fear	16	4.5
	Sadness	20	5.6
	Go to health facility	318	89.8

Do you have hepatitis B?	Yes	25	7
	No	329	92.9

Whom would you communicate to about your illness?	Physician	79	22.3
	Parents	132	37.3
	Husband	142	40.1
	No one	1	0.3

What will you do if you think that you have symptoms of hepatitis B?	Go to health facility	182	51.4
	Go to traditional healers	169	47.7
	Will not go anywhere	3	0.9

If you had symptoms of hepatitis B, at what stage would you go to the health facility?	As soon as I realized the symptoms	288	81.4
	After 2-4 weeks of the appearance of the symptoms	41	11.9
	Own treatment fails	23	6.5
	Will not go to health facility	2	0.6

How expensive do you think is the diagnosis and treatment of hepatitis B?	Cheap	16	4.5
	Free	12	33.9
	Moderately expensive	65	18.4
	Expensive	70	19.8
	I do not know	191	54

What worries you if you are diagnosed with hepatitis B?	Cost of treatment	29	8.2
	Fear of transmitting the disease to family members	66	18.6
	Fear of death	245	69.2
	Discrimination by the society	5	1.4
	Nothing to worry about	9	2.5

**Table 5:** Association of demographic characteristics and mean KAP scores among pregnant women attending ANC in the University of Gondar Comprehensive Specialized Hospital, Northwest Ethiopia, in 2018.

Variables	*N* (354)	Knowledge score (mean ± SD)	*P* value	Attitude score (mean ± SD)	*P* value	Practice score (mean ± SD)	*P* value
Age							
15-26	199	4 (2.6)	0.044	5.6 (1.6)	0.00	2.2 (1.3)	0.134
27-38	139	4.2 (2.5)		5.7 (1.9)	0.00	2.3 (1.4)	
39-49	16	4.3 (3.5)		6.3 (1.8)	0.065	2.1 (1.8)	
Residence							
Urban	271	4.5 (2.6)	0.00	5.7 (1.6)	0.00	2.4 (1.3)	0.00
Rural	83	2.9 (2.5)		5.8 (1.7)		1.5 (1.2)	
Marital status							
Single	29	3.9 (3.3)	0.058	4.8 (1.6)	0.073	1.4 (1.4)	0.2
Married	320	4.1 (2.6)		5.8 (1.6)	0.00	2.3 (1.3)	
Divorced	5	3.2 (1.8)		5.2 (1.1)	0.022	1.0 (1.0)	
Religion							
Orthodox	307	4.2 (2.7)	0.9	5.7 (1.6)	0.000	1.4 (1.4)	0.001
Muslim	39	3.5 (2.5)		5.6 (1.6)		2.3 (1.3)	
Protestant	8	3.4 (2.2)		6.4 (1.1)		1.0 (1.0)	
Educational status							
Unable to read and write	94	2.8 (2.4)	0.000	5.7 (1.7)	0.001	1.6 (1.2)	0.000
Able to read and write	4	3.5 (1.5)		5 (0.8)		2.3 (0.9)	
Elementary	99	3.8 (2.7)		5.8 (1.5)		1.9 (1.3)	
Secondary	79	4.8 (2.3)		5.5 (1.6)		2.6 (1.3)	
College and above	78	5.3 (2.4)		5.9 (1.8)		2.7 (1.4)	
Occupation							
Self-employed	56	3.9 (2.4)	0.074	5.7 (1.8)	0.015	2.3 (1.4)	0.001
Government	65	5.7 (2.4)		5.6 (1.7)		2.7 (1.4)	
Housewife	193	3.7 (2.5)		5.7 (1.6)		2.1 (1.3)	
Not employed	40	3.5 (2.9)		5.8 (1.6)		1.9 (1.5)	
Income/month							
<500	251	3.7 (2.6)	0.000	5.7 (1.6)	0.000	2.0 (1.3)	0.000
500-3000	80	5.0 (2.5)		5.6 (1.7)		2.5 (1.4)	
3001-500	18	5.3 (2.6)		5.8 (1.8)		3.3 (1.4)	
>5000	5	6.2 (2.2)		6.4 (1.5)		3.2 (1.1)	

**Table 6:** Logistic regressions between knowledge, practice responses, and sociodemographic characteristics of women attending ANC in the University of Gondar Comprehensive Specialized Hospital, Northwest Ethiopia, in 2018.

Variables	Knowledge		AOR 95% CI	*P* value	Practice		AOR 95% CI	*P* value
	Poor *N* (%)	Good *N* (%)			Poor *N* (%)	Good *N* (%)		
Age								
15-26	149 (42.1)	50 (14.1)	1.01 (0.3-3.3)	0.991	161 (45.5)	38 (10.7)	0.71 (0.2-0.2.3)	0.56
27-38	99 (28.0)	40 (11.3)	1.2 (0.36-3.98)	0.751	109 (30.8)	30 (8.5)	0.82 (0.3-2.7)	0.75
39-49	12 (3.4)	4 (1.1)			12 (3.4)	4 (1.1)		0.134
Residence								
Urban	190 (53.7)	81 (22.9)	2.3 (1.2-4.4)	0.012	204 (57.6)	67 (18.9)	5.1 (1.9-13.2)	0.012
Rural	70 (19.8)	13 (3.7)			78 (22)	5 (1.4)		
Marital status								
Single	20 (5.6)	9 (2.5)	0.99		26 (7.3)	3 (0.8)		0.99
Married	235 (66.4)	85 (24)	0.99		251 (70.9)	69 (19.5)		0.99
Divorced	5 (1.4)	0 (0)			5 (1.4)	0 (0)		
Religion								
Orthodox	222 (62.7)	85 (24)	1.1 (0.2-5.8)	0.86	246 (69.5)	61 (17.2)	0.74 (0.2-3.7)	0.72
Muslim	32 (9)	7 (2)	0.6 (0.1-3.9)	0.64	30 (8.5)	9 (2.5)	0.9 (0.2-5.3)	0.9
Protestant	6 (1.7)	2 (0.6)			6 (1.7)	2 (0.6)		
Educational status								
No formal education at all	80 (22.6)	14 (4)	0.3 (0.14-0.58)	0.001	87 (24.6)	7 (2)	0.1 (0.1-0.35)	0.000
Able to read and write	4 (1.1)	0 (0)		0.99	4 (1.1)	0 (0)		0.99
Elementary	74 (20.9)	25 (7.1)	0.5 (0.3-1.0)	0.06	83 (23.4)	16 (4.5)	0.34 (0.2-0.7)	0.003
Secondary	54 (15.3)	25 (7.1)	0.74 (0.4-1.4)	0.37	58 (16.4)	21 (5.9)	0.65 (0.3-1.3)	0.21
College and above	48 (13.6)	30 (8.5)			50 (14.1)	28 (7.9)		
Occupation								
Self-employed	44 (12.4)	12 (3.4)	0.72 (0.3-1.8)	0.49	43 (12.1)	13 (3.7)	0.9 (0.35-2.3)	0.84
Government	36 (10.2)	29 (8.2)	2.1 (0.9-4.9)	0.082	40 (11.3)	25 (7.1)	1.8 (0.8-4.4)	0.15
Housewife	151 (42.7)	42 (11.9)	0.7 (0.3-1.6)	0.43	169 (47.7)	24 (6.8)	0.4 (0.2-0.98)	0.45
Not employed	29 (8.2)	11 (3.1)			30 (8.5)	10 (2.8)		
Income/month								
<500	194 (54.8)	57 (16.1)	1.7 (1.0-3.0)	0.05	216 (61)	35 (9.9)	2.5 (1.4-4.5)	0.03
500-3000	53 (15)	27 (7.6)	2.7 (1-7.2)	0.04	57 (16.1)	23 (6.5)	9.6 (3.5-26.6)	0.000
3001-500	10 (2.8)	8 (2.3)	2.6 (0.4-13.9)	0.37	7 (2)	11 (3.1)	9.3 (1.5-57.4)	0.85
>5000	3 (0.8)	2 (0.6)			2 (0.6)	3 (0.8)		

## Data Availability

Data were collected from selected pregnant women attending the ANC clinic in the University of Gondar Comprehensive Specialized Hospital, Northwest Ethiopia, and registered on Microsoft Excel spreadsheet and can be made available when asked.

## Abbreviations

ANC: Antenatal care
Anti-HBc: Hepatitis B core antibody
Anti-HBs: Hepatitis B surface antibody
CSA: Central Statistical Agency
DNA: Deoxyribonucleic acid
GCMHS: Gondar College of Medicine and Health Science
GUH: Gondar University Hospital
HBcAg: Hepatitis B core antigen
HBeAg: Hepatitis B envelope antigen
HBIG: Hepatitis B immune globulin
HBsAg: Hepatitis B surface antigen
HBV: Hepatitis B virus
IGM: Immunoglobulin M
KAP: Knowledge, attitude, and practice
MTCT: Mother-to-child transmission
WHO: World Health Organization.

## References

[1] Liang T. J. (2009). Hepatitis B: the virus and disease. *Hepatology*.

[2] World Health Organization (2017). *Global hepatitis report 2017*.

[3] Hussain S. F., Ahmad S. R., Muslehuddin O. M., Muslehuddin H. M. (2016). Knowledge, attitude and practice regarding hepatitis B among medical students. *International Journal of Community Medicine and Public Health*.

[4] Abebe A., Nokes D. J., Dejene A., Enquselassie F., Messele T., Cutts F. T. (2003). Seroepidemiology of hepatitis B virus in Addis Ababa, Ethiopia: transmission patterns and vaccine control. *Epidemiology and Infection*.

[5] Barut H. S., Günal Ö., Göral A., Etikan I. (2011). Prevalence of hepatitis B virus infection in children of HBsAg positive parents. *Mikrobiyoloji Bülteni*.

[6] Muhammad A., Ibrahim B., Ramadan A. (2016). Knowledge, attitude and practice regarding hepatitis B infection among nurses in public hospitals of Niger State, Nigeria. *International Journal of tropical Disease & Health*.

[7] Han Z., Yin Y., Zhang Y. (2017). Knowledge of and attitudes towards hepatitis B and its transmission from mother to child among pregnant women in Guangdong Province, China. *PLoS One*.

[8] Ngaira J. A. M., Kimotho J., Mirigi I. (2016). Prevalence, awareness and risk factors associated with hepatitis B infection among pregnant women attending the antenatal clinic at Mbagathi District Hospital in Nairobi, Kenya. *The Pan African Medical Journal*.

[9] Panlilio A. L. C. D., Grohskopf L. A., Heneine W., Ross C. S. (2005). Updated US Public Health Service guidelines for the management of occupational exposures to HIV and recommendations for postexposure prophylaxis. Morbidity and Mortality Weekly Report. *Recommendations and Reports*.

[10] Sinha S., Kumar M. (2010). Pregnancy and chronic hepatitis B virus infection. *Hepatology Research*.

[11] Adjei C. A., Asamoah R., Atibila F., Ti-enkawol G. N., Ansah-Nyarko M. (2016). Mother-to-child transmission of hepatitis B: extent of knowledge of physicians and midwives in eastern region of Ghana. *BMC Public Health*.

[12] Gboeze A. J., Ezeonu P. O., Onoh R. C., Ukaegbe C. I., Nwali M. I. (2015). Knowledge and awareness of hepatitis B virus infection among pregnant women in Abakaliki Nigeria. *Journal of Hepatitis Research*.

[13] Dun-Dery F., Adokiya M. N., Walana W., Yirkyio E., Ziem J. B. (2017). Assessing the knowledge of expectant mothers on mother–to-child transmission of viral hepatitis B in Upper West region of Ghana. *BMC Infectious Diseases*.

[14] Fikremariam B. (2014). *Prevalence of hepatitis B surface antigen and KAP towards HBV infection, among pregnant women attending selected antenatal Clinics in Addis Ababa, Ethiopia*.

[15] Frambo A. A., Atashili J., Fon P., Ndumbe P. (2014). Prevalence of HBsAg and knowledge about hepatitis B in pregnancy in the Buea Health District, Cameroon: a cross-sectional study. *BMC Research Notes*.

[16] ul Haq N., Hassali M. A., Shafie A. A., Saleem F., Farooqui M., Aljadhey H. (2012). A cross sectional assessment of knowledge, attitude and practice towards hepatitis B among healthy population of Quetta, Pakistan. *BMC Public Health*.

[17] Hang Pham T. T., Le T. X., Nguyen D. T. (2019). Knowledge, attitudes and practices of hepatitis B prevention and immunization of pregnant women and mothers in northern Vietnam. *PLoS One*.

[18] Eguchi H., Wada K. (2013). Knowledge of HBV and HCV and individuals’ attitudes toward HBV- and HCV-infected colleagues: a national cross-sectional study among a working population in Japan. *PLoS One*.

[19] ul Haq N., Malik Z., Nasim A., Riaz S., Mohammad G., Azhar S. (2016). Knowledge, attitude and practice towards hepatitis B among medical college students of Quetta, Pakistan. *Value in Health*.

[20] Wah C. P., Hung S. S., Ka C. O., Hsi L. T., Yeung L. T. (2012). Awareness and knowledge of hepatitis B infection and prevention and the use of hepatitis B vaccination in the Hong Kong adult Chinese population. *Chinese Medical Journal*.

[21] Kamal R. (2015). *Knowledge, attitude and practice towards hepatitis B among the residents in Dhaandhoo island, Maldives*.

[22] Rahman M. A., Mannan S. R. (2010). The knowledge, attitude and practices regarding HBV infection of married women in the reproductive age group living in different districts of Bangladesh. *Medicine Today*.

[23] Islands S. (2008). *Second generation surveillance of antenatal women and youth*.

[24] Wu C. A., Lin S. Y., So S. K., Chang E. T. (2007). Hepatitis B and liver cancer knowledge and preventive practices among Asian Americans in the San Francisco Bay Area, California. *Asian Pacific Journal of Cancer Prevention*.

[25] Cheung J., Lee T. K., Teh C. Z., Wang C. Y. M., Kwan W. C. P., Yoshida E. M. (2005). Cross-sectional study of hepatitis B awareness among Chinese and Southeast Asian Canadians in the Vancouver-Richmond community. *Canadian Journal of Gastroenterology*.

